# Metabolomic Signatures of Treatment Response in Bladder Cancer

**DOI:** 10.3390/ijms242417543

**Published:** 2023-12-16

**Authors:** Tiago Vieira de Sousa, Paula Guedes de Pinho, Joana Pinto

**Affiliations:** 1 Associate Laboratory i4HB–Institute for Health and Bioeconomy, University of Porto, 4050-313 Porto, Portugal; up202204707@edu.ff.up.pt; 2 UCIBIO–Applied Molecular Biosciences Unit, Laboratory of Toxicology, Faculty of Pharmacy, University of Porto, Rua Jorge Viterbo Ferreira 228, 4050-313 Porto, Portugal

**Keywords:** bladder cancer, treatment response, metabolomics, biomarkers, personalized medicine

## Abstract

Bladder cancer (BC) stands as one of the most prevalent urological malignancies, with over 500 thousand newly diagnosed cases annually. Treatment decisions in BC depend on factors like the risk of recurrence, the type of tumor, and the stage of the disease. While standard therapeutic approaches encompass transurethral resection of the bladder tumor, radical cystectomy, and chemo- or immunotherapy, these methods exhibit limited efficacy in mitigating the aggressive and recurrent nature of bladder tumors. To overcome this challenge, it is crucial to develop innovative methods for monitoring and predicting treatment responses among patients with BC. Metabolomics is gaining recognition as a promising approach for discovering biomarkers. It has the potential to reveal metabolic disruptions that precisely reflect how BC patients respond to particular treatments, providing a revolutionary method to improve accuracy in monitoring and predicting outcomes. In this article, we present a comprehensive review of studies employing metabolomics approaches to investigate the metabolic responses associated with different treatment modalities for BC. The review encompasses an exploration of various models, samples, and analytical techniques applied in this context. Special emphasis is placed on the reported changes in metabolite levels derived from these studies, highlighting their potential as biomarkers for personalized medicine in BC.

## 1. Introduction

Omics approaches have played a crucial role in unraveling the intricate mechanisms of diseases within biological systems. Their use has increased significantly over the past decades. At first, genomic studies were widely used in discovering genetic variations associated with certain diseases and diverse treatment outcomes. In addition to genomics, other omics technologies such as epigenomics, transcriptomics, proteomics, and metabolomics have also proven to be highly valuable in deepening our knowledge of biochemical mechanisms [1]. Metabolomics has a significant advantage in this scenario as it represents the final stage in the omics cascade, enabling the closest correlation to the observed disease phenotype [2]. This omics approach employs high-throughput analytical techniques to measure the levels of low molecular weight molecules (known as metabolites) in biological samples offering a better understanding of the biochemical mechanisms and pathways associated with a diverse range of diseases [3,4].

The variations observed among individuals in their genome, proteome, and metabolome have a profound influence on how they respond to treatment and the achieved outcomes. Thus, the concept of personalized medicine is based on the idea that individuals’ unique characteristics require tailored interventions for their specific diseases [5]. This concept has been applied in cancer research, particularly in prostate, bladder, and breast cancers, where diverse clinical outcomes pose significant challenges in the effective treatment and management of these diseases [6,7]. Particularly for bladder cancer (BC), personalized medicine is crucial due to the inherent heterogeneity of the disease [8,9]. BC exhibits diverse molecular profiles and clinical behaviors among patients, making the uniform treatment approaches less effective. Moreover, lifelong BC surveillance is common due to its high recurrence, which increases costs and affects the quality of life of patients. [6].

Several studies have used omics to find biomarkers for personalized medicine in BC [6,10]. A proteomics study revealed a panel of cytokines to predict recurrence after intravesical immunotherapy in BC patients with 85.5% accuracy [11]. Genomic studies, for instance, have revealed that modifications of oncogenes such as *cyclin-dependent kinase (CDK)* and *fibroblast growth factor receptor (FGFR3)* serve as predictive biomarkers of response to their respective inhibitors [12,13]. Although metabolomics research in BC personalized medicine may be less prevalent compared to genomics, its potential is enormous since changes in metabolite levels can be used as biomarkers to predict drug responses [5,14]. This article aims to provide a comprehensive review of studies that investigate the metabolic responses to various BC treatment options using metabolomics approaches. The primary objective is to contribute to the development of a personalized medicine approach for the management of this disease. The review will encompass an exploration of different models, samples and analytical techniques employed in biomarker discovery within this context. Emphasis will be placed on the reported changes in metabolite levels from these studies, highlighting their potential as biomarkers. The ultimate goal is to underline the potential of these metabolites to predict drug responses, thereby enhancing the capacity to tailor treatments for optimal patient outcome.

## 2. Bladder Cancer

### 2.1. Epidemiology and Risk Factors

**Figure 1:**
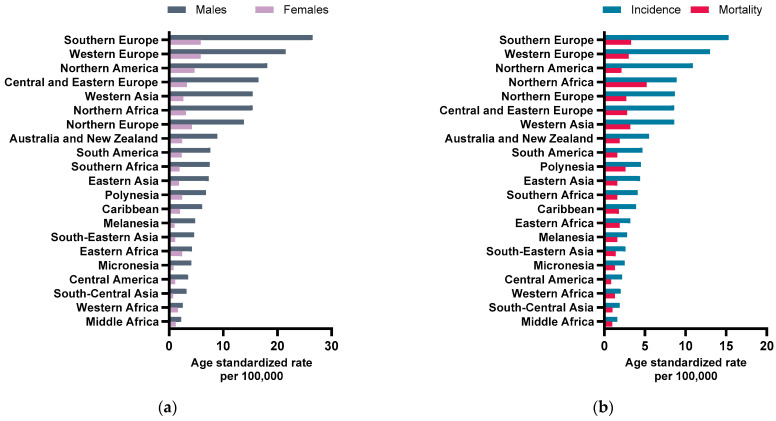
(**a**) Age-standardized (world) incidence rate of BC by sex; (**b**) age-standardized (world) incidence and mortality rates of BC. Data from December 2020 [20].

BC is the second most prevalent urological malignancy worldwide both in terms of newly diagnosed cases and associated deaths [15]. According to the International Agency for Research on Cancer, BC accounted for 573,278 new cases and 212,536 deaths globally in 2020, representing 3% and 2% of total reported cases and deaths, respectively [15]. Moreover, it exhibits notably higher incidence and mortality rates among men (Figure 1a), approximately four times more than in women, highlighting the presence of a cancer gender gap. Furthermore, the burden of this disease is more pronounced in developed countries (Figure 1b), particularly in southern and western Europe, which can be explained by the aging population and the higher prevalence of risk factors (e.g., tobacco smoking) [15,16]. The incidence and recurrent nature of BC impose a significant burden on healthcare systems, especially in less developed countries, where the number of cancer patients is expected to grow at a much faster rate [15,17]. The 5-year age-standardized survival rate of BC can be quite varied, ranging from 60% to 80% in Europe [18]. BC survival rates are greatly affected by the BC invasiveness. Roughly 75% of the newly diagnosed cases are non-muscle invasive BC (NMIBC) and patients have a reasonably high 5-year survival rate, while the remaining 25% are muscle invasive BC (MIBC), whose patients are expected to have a lower survival rate (around 60%) [19].

BC has several risk factors which can be separated into two categories: genetic susceptibility and external factors. Slow acetylator *NAT2 (N-acetyltransferase 2)* variants and *GSTM1 (glutathione S-transferase mu 1)* null genotypes are examples of possible BC genetic risk factors and, although they might not directly lead to BC, they may increase the susceptibility for external carcinogens such as tobacco smoke [17]. Indeed, tobacco smoking is one of the main risk factors for BC development and is also associated with a higher risk of BC progression and recurrence. It contains polycyclic aromatic hydrocarbons, aromatic amines and other known carcinogens that can cause DNA damage [19]. The fact that smoking is more common among men in developed countries shed light on the reasons behind the higher BC rates in these countries and the higher incidence among men [18]. Additional factors, such as exposure to occupational carcinogens, elevated levels of arsenic in drinking water, dietary habits, and certain medical conditions (e.g., schistosomiasis), have been linked to an increased risk of developing BC [19].

### 2.2. Histologic Variants, Stages and Molecular Subtypes

BC can be categorized based on the histological analysis of the tumor [21,22]. Urothelial cancer represents the predominant histological subtype in BC with approximately 25% of cases exhibiting variant histology within this carcinoma type. These variants may be associated with a more unfavorable prognosis when compared to classical urothelial carcinoma. Furthermore, non-urothelial BC subtypes, such as squamous cell carcinoma, sarcoma, and adenocarcinoma, contribute to the diverse histological spectrum of the disease. Urothelial carcinoma is commonly divided into NMIBC, which is restricted to the mucosa and submucosa of the bladder, and MIBC, which spreads to the muscularis propria of the bladder and can metastasize [23]. NMIBC tends to be clinically less aggressive but has high recurrence and progression and usually needs lifelong surveillance and management, while MIBC can have a rapid progression and spread to other organs and tissues like the lymph nodes, liver, and brain, consequently decreasing the patients’ long-term survival rates. Furthermore, NMIBC can manifest different identities, such as carcinoma in situ (CIS), papillary non-invasive tumors, and subepithelial connective tissue invasive tumors, with approximately 15% of NMIBC malignancies progressing to MIBC [24].

BC can be also classified based on its histopathological features, comprising low-grade (LG) and high-grade (HG) tumors [25,26]. Approximately half of the diagnosed BC tumors are categorized as LG, exhibiting a lower progression rate and yielding high survival rates for patients. In contrast, HG tumors possess a higher malignant potential and progression. LG tumors are typically categorized as NMIBC, while HG tumors can manifest as either NMIBC or MIBC.

The tumor, node, metastasis (TNM) classification serves as a crucial tool for delineating the stages of bladder cancer, enabling the identification of whether the cancer has infiltrated the bladder wall or initiated the spread to other tissues [27]. Table 1 succinctly outlines the key characteristics associated with each stage. NMIBC includes Ta, Tis, and T1 cases, whereas T2 to T4 are categorized as MIBC.

Finally, the considerable heterogeneity within BC has created a demand for the molecular subtype profiling of tumors. Large-scale mRNA (messenger RNA) expression profiling has been used in BC to identify different molecular subtypes [9] similar to other cancers. For instance, Kamoun et al. proposed a consensus classification of the various MIBC molecular subtypes [28]. They analyzed six different MIBC classifications and identified six molecular classes: luminal papillary (LumP), luminal non-specified (LumNS), luminal unstable (LumU), stroma-rich, basal/squamous (Ba/Sq), and neuroendocrine-like (NE-like). LumP tumors had strong *FGFR3* activity. LumNS were characterized by mutations in *ELF3 (E74 like ETS transcription factor 3)* and high stromal infiltration, while LumU tumors had an increased cell cycle activity and were associated with *TP53* mutations. Stroma-rich tumors also exhibited an increased presence of stromal and immune infiltrations. The *TP53* and *RB1 (Retinoblastoma 1)* genes were found to have the most common mutations in *Ba/Sq* and *NE*-like tumors. These mutations resulted in squamous differentiation and neuroendocrine differentiation, respectively.

The molecular subtype profiling can play a key role in identifying potential locations for therapeutic interventions. For example, *FGFR* inhibitors, like erdafitinib, are increasingly recognized as promising therapies for BC, benefiting patients with *FGFR3* overexpression, translocations, or mutations [28,29]. The implementation of a standardized classification system of BC molecular subtypes could significantly enhance the management of BC, leading to improved predictions of tumor outcomes and treatment responses.

### 2.3. Diagnosis and Prognosis

BC requires lifelong monitoring due to its high potential for recurrence and progression [30]. Thus, detecting this disease early is crucial for improving patient survival rates. Hematuria, dysuria, nocturia, and urine intermittency are symptoms that can be indicative of BC. Hematuria, in particular, is the symptom most frequently observed in cases of BC [31]. However, these symptoms are not exclusive to the disease and can be indicative of various other conditions, such as urinary tract infections. As a result, delayed detection of BC may occur, which can ultimately worsen the prognosis.

Commonly used methods for evaluating suspected BC patients include cystoscopy, urine cytology, ultrasound, computed tomography (CT), and magnetic resonance imaging (MRI) [32]. Cystoscopy, an invasive procedure, involves the insertion of a small tube into the bladder through the urethra to thoroughly examine and detect any potential abnormalities [33]. The sensitivity and specificity levels reported for cystoscopy range from 68 to 100% and 83 to 97%, respectively [33,34]. Urine cytology employs a microscope to detect cancer cells and other abnormal cells, providing a highly accurate diagnosis for patients in advanced stages of BC [35]. The sensitivity of cytology varies significantly at different stages with lower stages presenting lower sensitivity (4 to 31%). However, the overall sensitivity and specificity levels range from 13 to 86% and 73 to 100%, respectively [34]. Ultrasound can create a detailed image of the bladder and other organs. This allows for an accurate assessment of the tumor’s size and its potential to spread to other areas [36]. Meanwhile, the CT urogram provides valuable information about the tumor’s shape, size, and location within the bladder [36,37]. This procedure has sensitivity and specificity levels ranging from 46 to 87% and 78 to 100%, respectively [36,37]. On the other hand, the MRI urogram is exceptional at detecting soft tissue abnormalities, giving a clear indication of whether the tumor has extended to nearby tissues or lymph nodes [37,38]. This method has sensitivity and specificity levels ranging from 70 to 88% and 78 to 93%, respectively [34].

Although these diagnostic techniques are commonly used, they have several limitations [38,39,40]. Cystoscopy is invasive and can lead to potential misdiagnosis by failing to detect smaller tumors, inflammatory carcinoma, and carcinoma in situ (CIS). Urine cytology is non-invasive, but it lacks reliability due to false positive results. Furthermore, ultrasound exhibits limited accuracy, CT scans can miss flat lesions and CIS, and MRI procedures can be quite costly. Therefore, it is crucial to develop non-invasive techniques that provide improved sensitivity and specificity for diagnosing BC.

To improve diagnostic and follow-up strategies, urinary tumor biomarkers and other methods have been investigated, leading to the development of some FDA-approved tests, such as Bladder EpiCheck [41], Urovysion [42], nuclear matrix protein 22 (NMP22) [43], and bladder tumor antigen (BTA) [44]. Other procedures like UroSEEK [45], Xpert Detection [46], and CxBladder [47] also show promising performance but have not been approved by the FDA yet. However, these tests have not fully replaced the more specific and sensitive cystoscopy method, and some are difficult to implement in clinical practice due to high costs [10,48].

The discovery of more accurate prognosis markers to increase the chances of BC patient survival is also an unmet need. Currently, the assessment of the risk of recurrence is performed based on tumor characteristics and constitutes the basis for treatment and follow-up recommendations [32]. Some variants of urothelial carcinoma, such as micropapillary BC, and the presence of lymphovascular invasion are related to a worse prognosis [49,50]. Additionally, most LG NMIBC have a mutation in the FGFR3 gene that is associated with a better prognosis [48]. Other examples of possible prognosis markers are cell-free DNA, some non-coding RNA, and chloride intracellular channel 1 (CLIC1) [10,51].

### 2.4. Treatment and Management

BC treatment options vary depending on factors such as the stage and grade of the tumor, the risk of recurrence, age, and the patient’s overall health [32]. There are several methods for treating and managing BC, such as transurethral resection of the bladder tumor (TURBT), cystectomy, immunotherapy, and chemotherapy.

#### 2.4.1. TURBT and Radical Cystectomy

For the most common type of bladder tumors, NMIBC, treatment usually involves TURBT. This procedure can be used for both diagnostic and therapeutic purposes by using a resectoscope to cut the tumor into pieces and remove them through the urethra [52]. Examination of these tumor pieces helps ascertain the grade and stage of the malignancy while also relieving the patients’ symptoms when present [53]. The adverse effects of TURBT are generally limited, although hematuria and urinary tract infection are the most typical complications of the procedure [54]. Despite this, TURBT still presents significant limitations for the treatment of BC, namely incorrectly staging the tumor due to inadequate resection, the high risk of recurrence especially in higher grades of BC, the invasiveness of the surgery, and the risk of not detecting smaller malignancies that are not visible during the procedure [27,53]. Indeed, a second resection is recommended by the European Association of Urology for T1 and HG tumors due to their high likelihood of cancer progression [55]. It has been shown that performing a second resection before introducing the BCG therapy significantly decreases the risk of cancer recurrence [56]. Additionally, it could also detect residual tumors not previously found and decreases the risk of wrongly staging BC with 30% of T1 tumors being classified in a lower stage when the first TURBT was performed [57].

Radical cystectomy is a surgery often performed for MIBC tumors and some high-risk NMIBC malignancies. Moreover, it can also be preceded by neoadjuvant chemotherapy with cisplatin-based combinations to increase survival rates [58]. This procedure consists of surgically removing the bladder and nearby lymph nodes as well as removing part of the patient’s bowel so that the surgeon can perform a different procedure for urine to be stored and pass through [32]. Even though radical cystectomy increases survival rates in recommended patients when compared to TURBT, this invasive surgery has significant morbidity and mortality, particularly in older patients, where the mortality rates can reach 4% [59]. To decrease the high invasiveness of this surgery, a robotic-assisted radical cystectomy has gained attention since it is less invasive and decreases the need for blood transfusions because of smaller blood loss. This approach seems to have no additional complications or worse outcomes, the main disadvantage being the higher cost and longer duration of the surgery [60].

#### 2.4.2. Immunotherapy

One of the most common immunotherapy approaches performed for NMIBC is the Bacillus Calmette–Guérin (BCG) therapy. This intravesical therapy consists of further activating the immune system to kill cancer cells by using a weakened *mycobacterium* related to tuberculosis inserted into the bladder via a urethral catheter [61]. BCG is considered the golden standard for the treatment of high-risk NMIBC. Additionally, the use of this therapy after TURBT is proven to decrease recurrence rates and progression of the malignancy even when compared to TURBT and chemotherapy treatments [27,61].

Unfortunately, some patients have a weak response to this therapy and are usually indicated for a radical cystectomy. An increased level of programmed death ligand 1 (PD-L1) expression could be a biomarker for lack of response to BCG since PD-L1 can be beneficial for cancer cell growth by inducing T cell apoptosis [62]. In fact, finding programmed death 1 inhibitors is a promising way to select treatment for patients unresponsive to BCG. Pembrolizumab is a PD-1 inhibitor that has been approved by the FDA, while other drugs such as atezolizumab, durvalumab, avelumab and nivolumab are currently under study [63]. The combination of BCG with other immunotherapies has also been studied, namely the use of BCG with intravesical interferon-α (INF-α) and BCG with interlukin-2 (IL-2). They have shown good responses with INF-α, revealing better results when compared to patients who did not respond well to BCG alone, and IL-2 showing good tumor responses in animal models [64,65]. Furthermore, combining BCG with chemotherapy can also improve the treatment outcome, although it can have more toxic effects for the patient and, in some cases, may have worse results in decreasing the cancer recurrence [66,67].

#### 2.4.3. Chemotherapy

Chemotherapy is a widely used approach in managing BC, which is categorized into intravesical chemotherapy for NMIBC and systemic chemotherapy for MIBC [27,58]. Intravesical chemotherapy drugs include valrubicin, mitomycin C, epirubicin, pirarubicin, gemcitabine, and docetaxel [64,68]. Valrubicin is FDA approved for BCG-insensitive patients and CIS malignancy, but its efficacy is limited [64]. Mitomycin C is a drug that has cytotoxic properties through the crosslinking of DNA strands and can be used as an alternative to BCG [69]. Studies suggest that gemcitabine and docetaxel reduce disease recurrence, improve survival rates, and outperform BCG and mitomycin C [70,71]. Combining different drugs, like gemcitabine with docetaxel or with mitomycin C, has the potential to enhance treatment efficacy. Gemcitabine and docetaxel have shown promising response rates when given after TURBT [72]. Additionally, gemcitabine combined with mitomycin C is effective for treating HG NMIBC patients or those who are unresponsive to BCG [73]. Further research is needed to fully understand the effects of combination chemotherapy in BC treatment.

Systemic chemotherapy is categorized into neoadjuvant (before surgery) and adjuvant (after surgery) treatments [58]. Common platinum-based treatments include methotrexate, vinblastine, doxorubicin, cisplatin, and gemcitabine/cisplatin, showing potential for improving survival outcomes [74]. Adjuvant chemotherapy, recommended for patients not undergoing neoadjuvant treatment, may have less favorable results than neoadjuvant therapy but can delay BC progression and is essential for high-risk patients [75].

## 3. Tracking Treatment Response in BC Using Metabolomics Approaches

### 3.1. Metabolomics Workflow

Metabolomic approaches have been successful in detecting various types of cancer, including lung, breast, bladder, and prostate cancers [76,77,78,79]. One of the key advantages of metabolomics is that it considers environmental factors when analyzing cellular and systemic metabolic profiles. This positioning downstream in the omics cascade allows filling the gap between the genotype and phenotype for a more comprehensive understanding of these processes. Therefore, the molecules identified in a metabolomic analysis, including amino acids, lipids, nucleotides, and organic acids, among others, hold great promise as diagnostic, prognostic, and monitoring biomarkers [80,81]. These biomarkers can greatly assist in the personalized treatment and management of BC.

**Figure 2:**
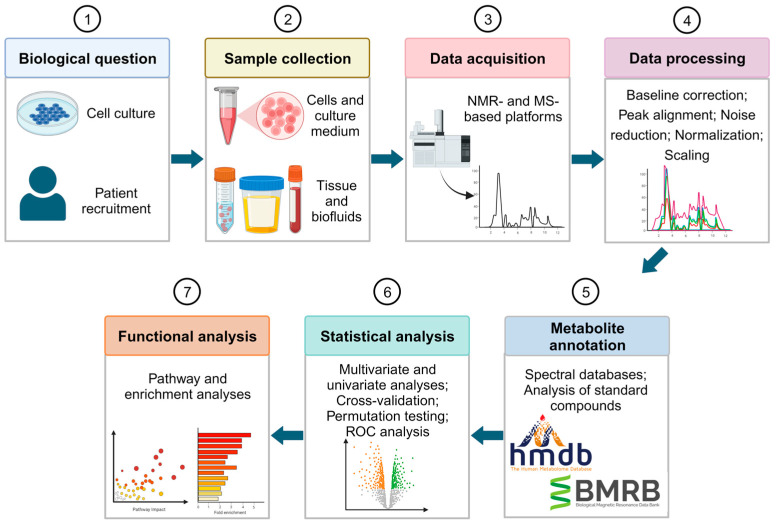
Overview of the untargeted metabolomics workflow for biomarker discovery in BC diagnosis and prognosis. Figure created using Biorender.com.

The metabolomics approach is commonly divided into untargeted and targeted analyses. The untargeted analysis consists of detecting all possible metabolites in a single analytical run, thereby providing a global metabolic profile of the samples, while the targeted analysis measures the concentrations of specific metabolites [82,83]. The latter can be used after untargeted analysis to obtain precise quantification of the compounds detected. Figure 2 shows the workflow designed for an untargeted metabolomic study to find biomarkers for BC diagnosis or prognosis. First, samples are collected to analyze their metabolic profile. Samples can be collected directly from the patient either as a biofluid, such as urine and blood plasma/serum, or as tissue. Additionally, studies can be based on in vitro models. These models are useful for an initial study of the metabolic changes associated with the desirable therapy, since it is less complex, and samples have less variability [84]. However, it may be difficult to extrapolate the findings of in vitro studies to in vivo systems. To achieve precise findings, the use of human tissues or biofluids is highly recommended. Moreover, biofluids have the added advantage of being non-invasive.

After sample collection, high-throughput analytical techniques are needed to detect and characterize the metabolites that are present in the samples. Mass spectrometry (MS) and nuclear magnetic resonance (NMR) spectroscopy stand out as the most used analytical platforms for metabolic profiling [82]. As detailed in Table 2, each technique presents distinct advantages and disadvantages, necessitating careful consideration in the study design. MS detects and quantifies the metabolites by measuring their mass via the mass-to-charge ratio (*m*/*z*). MS techniques are usually coupled to separation methods like gas chromatography (GC) and liquid chromatography (LC) to reduce sample complexity, enhance sensitivity, and facilitate metabolite annotation by providing a retention time identifier [85]. MS-based metabolomics studies require the use of quality control (QC) samples to assess and monitor the reliability, reproducibility, and overall quality of the analytical process [86]. In contrast, NMR relies on atomic nuclei absorption and re-emission, providing quantitative and reproducible measurements of known and unknown metabolites, requiring minimal sample preparation.

The main objective of data processing in metabolomics is to accurately identify and quantify the various features present in the data. The process involves several comparable steps for MS and NMR data including baseline correction, peak alignment, noise reduction, normalization, and scaling [82,87,88]. The endpoint of data processing is a feature matrix that includes intensities or abundances of relevant signals for each sample, representing the metabolic fingerprint of each individual.

Metabolite annotation is essential in metabolomics to identify and assign chemical identities to detected metabolomic features [89]. The most used method involves matching experimental data with reference data from databases or spectral libraries [90]. However, additional procedures are required to obtain accurate metabolite annotation. Several researchers have focused on minimal reporting standards for levels of confidence in metabolite annotation [91], which are essential for accurately evaluating the credibility of any biochemical interpretation within a metabolomics study. To validate metabolite annotations, it is recommended to use at least two independent and complementary datasets for the analysis of a chemical reference standard with suspected structural equivalence. Datasets may consist of retention time/index and mass spectrum data, accurate mass and tandem MS (MS/MS) results, as well as full ^1^H and/or ^13^C NMR data. All analyses should be carried out under identical analytical conditions in the same laboratory.

**Table 2:** Advantages and disadvantages of MS- and NMR-based metabolomic techniques [92,93,94].

Techniques	Advantages	Disadvantages
GC-MS	High resolution High sensitivity High accuracy High repeatability High discrimination between similar molecules	Ineffective on thermolabile compounds Needs derivatization of non-volatile metabolites Slightly expensive
LC-MS	Good resolution High sensitivity Effective on thermolabile compounds	Lack of standard spectral libraries Adducts formation Long analytical time Slightly expensive
NMR	Non-destructive analysis High reproducibility Relatively simple sample preparation Highly quantitative Relatively fast	Low sensitivity Peak overlapping Very expensive

Statistical methods are employed to find metabolites that are significantly expressed, which are categorized into multivariate and univariate analyses. Multivariate analysis identifies patterns and differences between case and control groups considering a large number of variables. Some examples of multivariate methods include principal component analysis (PCA), and partial least square-discriminant analysis (PLS-DA) [95]. Cross-validation and permutation testing are important in assessing the robustness of predictive models, particularly in the case of small sample sizes [96,97]. Conversely, univariate analysis such as *t*-tests, and analysis of variance (ANOVA) evaluate a single variable. Given the presence of multiple metabolites in biological samples, multiple testing corrections (e.g., Bonferroni, false discovery rate) are essential to mitigate false positives associated with univariate testing [98]. In addition, biomarker discovery studies also consider receiver operator characteristic (ROC) curve analysis to evaluate the performance of biomarkers in terms of sensitivity, specificity, and accuracy [99].

Functional analysis is the next step of a metabolomics study. The compounds detected in this analysis are linked to their biological pathways and functions to uncover altered biomarkers and their associations with biological and medical conditions [98]. Enrichment or pathway analyses are common methods employed for this purpose [100].

Ultimately, the study’s findings need validation in larger cohorts, considering training and testing sets for application in clinical practice for diagnostic, prognostic, and monitoring purposes [101]. Integration with other omics data, including genomics, transcriptomics, or proteomics, further enhances the understanding of the functions and interactions of differentially expressed metabolites [102].

### 3.2. Metabolomic Biomarkers of Treatment Response in BC

Metabolomics has the potential to predict disease progression and treatment response by monitoring metabolic changes. Thus, discovering biomarkers through metabolomics for personalized BC treatment is of great importance. This approach has the potential to decrease the impact of the illness, as it is less invasive and more cost-effective. This section presents an extensive review of studies that have used metabolomics approaches to identify potential biomarkers of treatment response in BC. The search was conducted on the PubMed database with the specified keywords: (“metabolomics” OR “metabolic profiling”) AND (transurethral resection OR chemotherapy OR immunotherapy) AND “bladder cancer”. The search included all English publications without any restrictions on the publication date and was conducted in May 2023. The search retrieved 35 papers that were screened according to the following inclusion criteria: (1) papers reporting original results; (2) papers using metabolomic approaches; (3) papers focused on current BC treatment options (transurethral resection of the bladder tumor, chemotherapy, and immunotherapy). After excluding reviews and other studies not relevant to the topic, seven studies were considered eligible, from which three were in vitro studies (Table 3), three considered human biofluids (blood serum and urine), and one considered tumoral and non-tumoral tissues (Table 4).

#### 3.2.1. In Vitro Studies

MIBC patients often receive cisplatin-based chemotherapy for treatment. The primary mechanism of action of cisplatin is through DNA damage, which activates several signal transduction pathways leading to apoptosis [103]. Resistance to cisplatin chemotherapy is a major limitation, but the reasons behind this resistance remain mostly unknown [104]. Two in vitro studies have been conducted to analyze the metabolism of cisplatin-resistant BC cells using metabolomics [105,106]. Another in vitro study delved into the metabolic mechanisms driving the multiple drug resistance in BC [107]. Table 3 provides a summary of the experimental design and key findings from these studies.

Lee et al. focused on lipid metabolism to investigate the mechanisms and pathways underlying the resistance to cisplatin-based treatments [106]. Changes in lipid metabolism have been associated with aggressive forms of some cancers and may be used as potential cancer biomarkers [108]. For this, the authors used two transitional cell carcinoma (T24) cell lines, one cisplatin-sensitive (T24S) and one cisplatin-resistant (T24R). Through ultra-performance liquid chromatography-mass spectrometry (UPLC-MS), differentially expressed lipids were identified by comparing the T24S and T24R cells [106]. The lipid profiles of T24S and T24R cells treated with and without an acetyl-CoA synthetase 2 (ACSS2) inhibitor were also examined. The purpose was to investigate the significance of this enzyme in fatty acid synthesis by analyzing T24S+ and T24R+ cells (treated with the inhibitor) in comparison to T24S− and T24R− cells (untreated). The results revealed a decrease in cisplatin-induced apoptosis and an increase in lipidic production in T24R cells, suggesting a link between the two events. Compared to T24S cells, the T24R cells displayed a high number of downregulated lipid species, including various phosphatidylethanolamines (PE) and triglycerides (TG). On the other hand, one ceramide (CE) and several phosphatidylcholines (PCs) and sphingomyelins (SMs) were found to be upregulated in T24R cells. ACSS2’s association with treatment resistance was confirmed by the authors, who found greater differences between T24S+ and T24R+ cells compared to the differences observed between T24S− and T24R− cells. ACSS2 inhibition did not significantly affect most T24R-specific lipidic metabolites, although metabolites such as CE (18:1), CE (22:6), TG (49:1), and TG (53:2) were greatly affected by the ACSS2 inhibitor. These results suggest an association between cisplatin resistance and altered lipid metabolism. The authors highlighted the potential of modulating lipid metabolism as a promising approach to combat cisplatin resistance.

Wen et al. also used T24S and T24R cell lines in their study, but instead of using an MS-based technique, they performed a real-time NMR-based metabolomics approach to detect metabolic changes in cisplatin-resistant BC cells [105]. In this approach, the metabolites generated from ^13^C-glucose tracer were monitored by two-dimensional (2D) ^1^H-^13^C Heteronuclear Single Quantum Coherence (HSQC) NMR in real time. A Western blot analysis was also performed to check possible alterations of fatty acid metabolism involving acetate. The T24R cells revealed higher glucose consumption, higher and faster acetate accumulation, higher levels of fatty acids and lower production and excretion of lactate. On the other hand, T24S cells showed higher and faster lactate and alanine accumulation. Thus, cisplatin resistance may be related to glucose consumption and acetate production. The results from the Western blot revealed an increase in upstream metabolic regulators in T24R cells, namely phosphorylated epidermal growth factor receptor (EGFR) and mammalian target of rapamycin (mTOR), suggesting an association of these regulators with cisplatin resistance. In addition, acetyl-CoA carboxylase and ACSS2 were more highly expressed in T24R cells. The higher levels of both enzymes may indicate that T24R cells have an enhanced fatty acid synthesis via two carbon metabolism involving acetate. Moreover, the authors confirmed that glucose-derived endogenous acetate contributes to the enhanced fatty acid de novo synthesis in T24R cells. Inhibition of the ACSS2 pathway decreased the de novo synthesis of fatty acids in T24R cells only. ACSS2 expression in bladder tumor tissues from patients receiving cisplatin-based chemotherapy was further increased, confirming the relevance of ACSS2 in cisplatin resistance.

Zhu et al. aimed to investigate the mechanism of multiple drug resistance in BC with a specific focus on exploring the involvement of both c-MYC and polyamine metabolism [107]. Polyamines, as essential low molecular compounds, play a critical role in eukaryotic cell growth and function. In contrast, c-MYC is a well-known oncogene that significantly influences cell proliferation, senescence, and apoptosis [107,109,110]. The authors created a pirarubicin-resistant cell line (T24/THP) with multiple drug resistance by gradually exposing the T24 cell line to increasing concentrations of pirarubicin, epirubicin, and mitomycin C. This resistant cell line was compared with the parental T24 cell line. To gain insights into the underlying mechanisms of multiple drug resistance, a combination of untargeted metabolomics analysis and gene microarray detection was employed. The researchers discovered variations in the concentrations of numerous metabolites that are associated with the metabolism of arginine and proline. These findings indicate that this pathway could potentially play a significant role in the development of resistance. Moreover, the levels of two polyamines (putrescine and spermidine), as well as c-MYC, were observed to decrease in pirarubicin-resistant cells. Although the expression of ornithine decarboxylase (ODC1) and spermidine synthetase (SRM) is low in resistant cells, their knockdown surprisingly revealed contrasting outcomes by enhancing treatment sensitivity. ODC1 and SRM may contribute to drug resistance, as their lower expression in pirarubicin-resistant cells could be influenced by other upstream genes. Furthermore, the regulation of ODC1 and SRM expression by c-MYC, as well as its impact on drug resistance in T24PR cells, remains a subject of debate due to conflicting results from different studies on the therapeutic benefits of its downregulation [111,112].

Overall, these studies provide novel insights into the mechanisms of drug resistance in BC, offering potential targets for future therapeutic interventions. The three studies examined revealed a notable challenge in achieving reliable metabolite annotation, which is particularly evident in LC-MS investigations featuring numerous differential metabolites. This limitation poses a potential obstacle to the precise identification of critical biomarkers or therapeutic targets. Furthermore, a primary concern in these studies is the inherent challenge of extrapolating results to in vivo models, suggesting that the identified potential biomarkers (differential metabolites) may not comprehensively reflect the complex physiological processes within the human body. Despite this limitation, in vitro studies play a vital role in providing essential insights into the metabolic pathways and treatment-induced alterations specific to bladder tumor cells.

**Table 3:** Metabolomic studies investigating the metabolic dysregulations associated with therapy resistance in BC cell lines.

Cell Lines	Treatment	Study Groups	Analytical Techniques	Main Results	References
Transitional Cell Carcinoma (T24)	Cisplatin (chemotherapy)	T24S: Cisplatin-sensitive T24 T24R: Cisplatin-resistant T24 T24S−: Cisplatin-sensitive without ACSS2 inhibitor T24 S+: Cisplatin-sensitive with ACSS2 inhibitor T24R−: Cisplatin-resistant without ACSS2 inhibitor T24 R+: Cisplatin-resistant with ACSS2 inhibitor	UPLC-MS	T24R vs. T24S: ↑ CE (22:6); PC (35:4); PC (36:6); SM (d42:2); SM (d42:3); SM (d32:1) ↓ PE (*p*-36:4)/PE (*o*-36:5); PE (*p*-38:4)/PE (*o*-38:5); PE (*p*-40:5)/PE (*o*-40:6); TG (48:0); TG (49:0); TG (49:0); TG (49:1); TG (50:0); TG (52:0); TG (54:5) T24S+ vs. T24S− and T24R+ vs. T24R−: ↓ CE (22:6); CE (18:1); TG (49:1); TG (53:2)	Lee et al., 2018 [106]
Transitional Cell Carcinoma (T24)	Cisplatin (chemotherapy)	T24S: Cisplatin-sensitive T24 T24R: Cisplatin-resistant T24	2D NMR (^1^H-^13^C Heteronuclear Single Quantum Coherence)	T24R cells exhibited: Higher glucose consumption Higher and faster acetate accumulation Higher levels of fatty acids Lower production and excretion of lactate T24S cells exhibited: Higher and faster lactate and alanine accumulation	Wen et al., 2019 [105]
Transitional Cell Carcinoma (T24)	Pirarubicin (chemotherapy)	T24: Parental T24 T24/THP: Pirarubicin-resistant T24	LC-MS	T24/THP vs. T24: Dysregulations in the levels of more than 200 metabolites (annotation not provided), including lower levels of putrescine and spermidine Arginine and proline metabolism pathway showed the strongest correlation with the differential metabolites	Zhu et al., 2022 [107]

2D NMR: two-dimensional nuclear magnetic resonance spectroscopy; ACSS2: acetyl-CoA synthetase 2; CE: ceramides; LC-MS: liquid chromatography-mass spectrometry; PC: phosphatidylcholine; PE: phosphatidylethanolamide; SM: sphingomyelin; TG: triglycerides; UPLC-MS: ultra-performance liquid chromatography-mass spectrometry; ↑ increased levels; ↓ decreased levels.

#### 3.2.2. Human Biofluid and Tissue Studies

Investigating the metabolomic features of cancer treatment response in human subjects is crucial for enhancing our knowledge of therapeutic outcomes and improving personalized treatment strategies. However, the number of reported studies in this field for BC is relatively limited. Our search yielded two studies that investigated the impact of TURBT on the metabolic profile characteristic of BC using human biofluids (blood serum and urine) [113,114]. Additionally, another study focused on evaluating the impact of submucosal injection of gemcitabine prior to TURBT on both BC and adjacent normal tissue [115], while a separate investigation aimed to identify biomarkers in blood serum for predicting the effectiveness of neoadjuvant chemotherapy in BC [116]. Table 4 provides a concise overview of the biological samples, cases under study, analytical techniques and main findings found in these studies.

Bansal et al. previously reported that alterations in the levels of dimethylamine (DMA), malonate, lactate, glutamine, histidine, and valine demonstrate a 95% detection rate for BC cases when compared to healthy controls [117]. The same research team carried out a targeted metabolomics analysis to examine the levels of the aforementioned metabolites and their potential to differentiate between pre- and post-operative conditions in patients with BC [113]. The study consisted of 130 men who were diagnosed with BC (LG *n* = 33–35 and HG *n* = 31) and underwent TURBT. Blood serum samples were collected before surgery (pre-operative samples) and 30, 60, and 90 days after surgery (post-operative samples). To evaluate the expression level of potential biomarkers, the authors conducted a comparison between pre- and post-operative sample signatures, which were further divided into LG and HG categories. The study also included an additional group of 52 controls. Of the selected metabolites, DMA, lactate, glutamine, histidine, and valine levels were higher in LG BC compared to controls, and these levels decreased after TURBT. Moreover, DMA, malonate, lactate, histidine, and valine also presented higher levels in HG BC compared to controls and decreased levels throughout post-operation time. These findings suggest that DMA, lactate, histidine, and valine hold promise as potential non-invasive biomarkers for inclusion in follow-up protocols for BC.

Previous research conducted by Jacyna et al. highlighted changes in the levels of various metabolites involved in amino acid, pyrimidine, purine, and energy metabolisms in the urine of BC patients in comparison to that of healthy controls [118]. Later, the authors applied the same multi-platform metabolomics approach to measure levels of selected metabolites in the urine of BC patients who had undergone TURBT [114]. Eight men and two women diagnosed with NMIBC participated in this study. The samples were collected before, the day after, and two weeks after the TURBT procedure. Two weeks after TURBT, the levels of several urinary metabolites were found significantly decreased in comparison with pre-TURBT, including metabolites participating in the amino acid, purine, and pyrimidine metabolisms. These molecules participate in a wide range of biological processes such as cell proliferation and tumor progression [119,120]. In addition, several differential molecules found by Jacyna et al. are methylated metabolites possibly related with processes of DNA methylation enhanced during BC [121]. In particular, the levels of 1,3-dimethyluracil, N1-methyl-2-1,3-dimethyluracil, and methylnicotinamide were significantly decreased two weeks after TURBT in comparison to pre-TURBT. These results indicate that post-surgery metabolomic-based approaches can effectively assess the eradication of the tumor’s metabolic phenotype. The study faced a significant limitation due to the small number of samples, as only 20% of the initially recruited patients attended the follow-up visit. Further research is required to precisely identify biomarkers that can be implemented in clinical settings.

**Table 4:** Metabolomic studies investigating the metabolomic dysregulations associated with BC treatment in human tissues and biofluids.

Biological Matrix	Treatment	Study Groups	Analytical Techniques	Main Results	References
Serum	TURBT (surgery)	Pre-operative: LG, *n* = 35; HG, *n* = 31 Post-operative: LG, *n* = 33; HG, *n* = 31 Controls, *n* = 52	^1^H NMR	Post- vs. pre-operative LG: ↓ DMA, lactate, glutamine, histidine, valine Post- vs. pre-operative HG: ↓ DMA, lactate, histidine, valine, malonate The levels of DMA, glutamine and malonate are similar to those of controls at 90 days after surgery	Gupta et al., 2020 [113]
Urine	TURBT (surgery)	NMIBC patients: Pre-TURBT, *n* = 10 Post-TURBT, *n* = 10	HPLC-MS GC-MS	Post- (2 weeks) vs. pre-TURBT: ↓ N-Acetylneuraminic acid, androsterone 3-glucuronide, creatine riboside, creatinine, 5,6-dihydrouridine, N6,N6-dimethyl-lysine, 1,3-dimethyluracil, glucosylgalactosyl hydroxylysine, glutarylcarnitine, guanidinosuccinic acid, indolelactic acid, indoxyl sulfate, N6-methyladenosine, 3-methylglutarylcarnitine, 1-methylguanine, 1-methylinosine, N6-methyl-lysine, succinylcarnitine, N-methylnicotinamide, N-methylnicotinamide, glutamic acid, O-sebacoylcarnitine, succinyladenosine, tryptophan, valine	Jacyna et al., 2022 [114]
Bladder tissue	Gemcitabine (chemotherapy)	Pre-gemcitabine: BC, n = 12 (Ta *n* = 2, T1 *n* = 1, and T2 *n* = 9); normal, *n* = 12 Post-gemcitabine: BC, n = 12 (Ta *n* = 2, T1 *n* = 1, and T2 *n* = 9); normal, *n* = 12	UPLC-MS	Among the 34 BC-associated metabolites (pre-gemcitabine BC vs. pre-gemcitabine normal), the levels of bilirubin and retinal recovered after gemcitabine treatment (post-gemcitabine BC vs. pre-gemcitabine normal) Histamine (↑) and thiamine (↓) levels found altered in adjacent normal tissue after gemcitabine treatment	Yang et al., 2019 [115]
Serum	Gemcitabine and cisplatin (neoadjuvant chemotherapy)	MIBC patients: NAC-sensitive, *n* = 6 (T2 *n* = 4, and T3 *n* = 2) NAC-resistant, *n* = 12 (T2 *n* = 5, T3 *n* = 6, and T4 *n*= 1)	^1^H NMR UPLC-MS	NAC-sensitive vs. NAC-resistant: ↑ Glutamine, taurine ↓ 2-Hydroxy-3-methylvalerate, 3-methyl-2-oxovalerate, 3-hydroxybutyrate, alanine, glutamate, pyruvate, pyroglutamate, glycine, hypoxanthine	Zhuang et al., 2022 [116]

^1^H NMR: proton nuclear magnetic resonance spectroscopy; BC: bladder cancer; DMA: dimethylamine; GC-MS: gas chromatography-mass spectrometry; HG: high grade; HPLC-MS: high performance liquid chromatography-mass spectrometry; LG: low grade; MIBC: muscle invasive BC; NAC: neoadjuvant chemotherapy; NMIBC: non-muscle invasive BC; TURBT: transurethral resection of bladder tumor; UPLC-MS: ultra-performance liquid chromatography-mass spectrometry.; ↑ increased levels; ↓ decreased levels.

The submucosal injection of gemcitabine prior to TURBT may prevent BC recurrence, but the underlying mechanism remains unknown. In this context, Yang et al. applied a MS-based metabolomic approach to analyze metabolic changes in BC and normal bladder tissues before and after treatment with gemcitabine [115]. The study participants comprised a small cohort of 12 BC patients (nine men and three women), all of whom had undergone TURBT. Almost all patients were diagnosed with HG, T2 stage tumors (*n* = 9), except one T1 BC female patient, and two male patients were diagnosed with Ta malignancies. First, the authors compared pre-gemcitabine normal tissues with pre-gemcitabine BC tissues indicating that changes in glutathione, purine, and thiamine metabolism pathways were significantly associated with BC malignancy. After gemcitabine treatment, the bilirubin and retinal levels found higher in BC tissues recovered to levels similar to that of pre-gemcitabine normal tissue. This result suggested that these metabolites may be the potential targets of gemcitabine for reducing BC recurrence. The submucosal injection of gemcitabine also affected the metabolic composition of the adjacent normal tissue since higher levels of histamine and lower levels of thiamine were observed in post- compared with pre-gemcitabine normal tissues. Yang et al. suggested that histamine could protect against disease relapse whereas thiamine could be related to side effects of the treatment.

Zhuang et al. employed NMR- and MS-based metabolomics methods to discover metabolic biomarkers for predicting the effectiveness of neoadjuvant chemotherapy (NAC) in MIBC patients [116]. Before the first chemotherapy, blood serum was collected from 18 patients who were scheduled to receive NAC (gemcitabine and cisplatin). The patients were divided into two groups: six patients were identified as NAC-sensitive (T2 *n* = 4, and T3 *n* = 2), while the remaining 12 patients were categorized as NAC-resistant (T2 *n* = 5, T3 *n* = 6, and T4 *n* = 1). The analysis of serum samples was performed using both ^1^H NMR spectroscopy and UPLC-MS. The sera of NAC-sensitive patients displayed decreased levels of various metabolites, such as organic acids and amino acids, in contrast to NAC-resistant patients. Furthermore, there was an upregulation of glutamine and taurine in NAC-sensitive patients as opposed to NAC-resistant patients. From these metabolites, the authors emphasized the variations found in the levels of glutamine, taurine, glycine, and hypoxanthine, which were detected in both analytical methods with the same trend. The pathway analysis unveiled an enrichment of glutathione metabolism, glycine, serine and threonine metabolism as well as glyoxylic acid and dicarboxylic acid metabolism. The three putative metabolic pathways may be important for determining chemotherapy sensitivity. Although this study had limitations in terms of the number of participants, it successfully demonstrated that there are variations in metabolic phenotypes between NAC-sensitive and NAC-resistant patients. The discovery highlights the potential of metabolomics in customizing medical treatments for individuals diagnosed with MIBC.

Several limitations in the reviewed studies should be highlighted, as they hinder the robustness of treatment response biomarkers in BC. These limitations included small sample sizes, lack of robustness in metabolite annotation (mostly level 2 [91]), and some studies’ failure to consider both genders. The limited sample sizes may arise from challenges in patient recruitment, as certain studies involved multiple time points gathered from each participant. Moreover, the diverse range of BCa tumors, which included different stages and grades, along with diverse treatment protocols and research methodologies, pose challenges when attempting to compare different studies.

## 4. Conclusions

The application of metabolomics to discover biomarkers that indicate response to BC treatments is still in its early stages. Preliminary studies have shown that different treatments have a noticeable impact on the metabolic profiles of cells, biofluids (serum and urine) and bladder tissue. Initial investigations revealed that cisplatin resistance in bladder tumor cells is associated with changes in lipid metabolism, increased glucose consumption, and elevated acetate production. Conversely, resistance to multiple chemotherapy drugs in bladder tumor cells has been associated with dysregulations in arginine and proline metabolism. Human samples analysis showed that the metabolic profile characteristic of BC in biofluids and tumor tissue reverts to a profile resembling that of the cancer-free group after TURBT and submucosal injection of gemcitabine before TURBT. Moreover, patients exhibiting sensitivity to neoadjuvant therapy displayed elevated levels of glutamine and taurine, along with decreased levels of glycine and hypoxanthine, in their serum metabolic profiles prior to treatment. These findings highlight the remarkable potential of metabolomics in unravelling the complex metabolic shifts associated with responses to BC treatment. To advance in this field, it is crucial to validate in vitro findings in in vivo models, increase the number of participants in human studies, and improve the accuracy of metabolite annotation. Moreover, integrating metabolomics data with other omics and artificial intelligence has the potential to enhance the precision of predicting treatment outcomes in BC.

## Figures and Tables

**Table 1:** TNM classification of BC according to the European Association of Urology Guidelines [27].

Primary Tumor (T)	
TX	Primary tumor cannot be assessed
T0	No evidence of tumor
Ta	Non-invasive papillary carcinoma
Tis	Carcinoma in situ (CIS)
T1	Tumor invades subepithelial connective tissue
T2	Tumor invades muscle
T2a	Tumor invades superficial muscle
T2b	Tumor invades deep muscle
T3	Tumor invades perivesical tissue
T3a	Microscopically
T3b	Macroscopically
T4	Tumor invades other tissues
T4a	Tumor invades prostate stroma, seminal vesicles, uterus, or vagina
T4b	Tumor invades pelvic or abdominal wall
Regional nymph nodes (N)	
NX	Regional lymph nodes cannot be assessed
N0	Regional lymph nodes without metastasis
N1	Metastasis in a single lymph node in the true pelvis
N2	Metastasis in multiple regional lymph nodes in the true pelvis
N3	Metastasis in common iliac lymph nodes
Distant metastasis (M)	
M0	No distant metastasis
M1a	Non-regional lymph nodes
M1b	Other distant metastasis

## Data Availability

Not applicable.
